# *Bacillus* Bio-Organic Fertilizer Altered Soil Microorganisms and Improved Yield and Quality of Radish (*Raphanus sativus* L.)

**DOI:** 10.3390/plants14091389

**Published:** 2025-05-05

**Authors:** Yingbin Qi, Zhen Wu, Yachen Wang, Rong Zhou, Liwang Liu, Yan Wang, Jiying Zhao, Fangling Jiang

**Affiliations:** 1 College of Horticulture Science and Technology, Hebei Normal University of Science and Technology, Qinhuangdao 066004, China; shuaibin678@163.com; 2 Key Laboratory of Biology and Germplasm Enhancement of Horticultural Crops in East China, College of Horticulture, Nanjing Agricultural University, Ministry of Agriculture, Nanjing 210095, China; wzh@njau.edu.cn (Z.W.); 2014104060@njau.edu.cn (Y.W.); 2021002@njau.edu.cn (R.Z.); nauliulw@njau.edu.cn (L.L.); wangyanhs@njau.edu.cn (Y.W.); 2018804169@njau.edu.cn (J.Z.); 3 Hebei Key Laboratory of Horticultural Germplasm Excavation and Innovative Utilization, Qinhuangdao 066600, China; 4 Department of Food Science, Aarhus University, DK-8200 Aarhus, Denmark

**Keywords:** radish, bio-organic fertilizer, rhizosphere soil, soil microbial community composition

## Abstract

Excessive use of fertilizers will not only cause the enrichment of soil N nutrients, soil secondary salinization, soil acidification, and an imbalance of the soil microbial community structure, but will also lead to the nitrate content of vegetables and the ground water exceeding the standard. The application of bio-organic fertilizer could reduce the amount of mineral fertilizer used. However, the effects of nitrogen reduced with different bio-organic fertilizers on soil chemical properties, microbial community structure, and the yield and quality of radish are not clear. In a field experiment, we designed six fertilization treatments: no fertilization (CK), conventional fertilization (T1), a total nitrogen reduction of 20% (T2), and a total nitrogen reduction of 20% with “No. 1”, “Seek” or “Jiajiapei” bio-organic fertilizers. The results showed that nitrogen reduction of 20% with *Bacillus* bio-organic fertilizer (N1) significantly increased the organic matter, pH, total nitrogen content, and the relative abundance of *Bacillus* and *Streptomyce* in the soil compared with T1. RDA analysis showed that the pH, organic matter content, invertase and fluorescein diacetate enzyme activity of the soil were significantly correlated with the soil microbial community structure. In addition, the yield and Vc content in radish were increased with the application of bio-organic fertilizers, while on the contrary, the nitrate and cellulose content were decreased, and the N1 treatment showed the best effect. Moreover, the yield had a significant positive correlation with *Bacillus*. Overall, nitrogen reduction with bio-organic fertilizers, especially full-effective “No. 1” enriched with *Bacillus*, could alter the soil microbial community structure and effectively improve soil fertility, which in turn enhanced the yield and quality of radish. An application of *Bacillus* bio-organic fertilizer was an effective strategy to improve soil quality and vegetable safety.

## 1. Introduction

Synthetic fertilizer is almost always needed to promote a crop’s yield and quality [1,2]. However, the application of chemical fertilizers might be greater than the amount of fertilizer required by crops [3]. Excessive and unreasonable application of fertilizers leads to lower fertilizer use efficiency, degradation of soil quality, decline of vegetable quality, and pollution of the ecological environment [4,5,6,7]. Thus, for sustainable development, it is imperative to seek a method of nitrogen fertilizer management that reduces chemical fertilizer application while stabilizing the yield and quality of vegetables. Reasonable technology for partial substitution of chemical fertilizer is of great significance for reducing environmental pollution and ensuring high yield and quality of vegetables, which is also crucial for promoting beneficial rhizosphere interactions for sustainable agricultural production [8].

Reduction of nitrogen fertilizer and simultaneous application of bio-organic fertilizer is recognized as the most effective N fertilizer management practice for substituting organic or chemical fertilizer alone [9]. Previous research found that mineral fertilizer combined with bio-organic fertilizer could maintain the balance of the soil microbial community by altering the soil pH and soil nutrition content [10]. Soil microbial diversity is critical for soil health [11], and soil microorganisms play a vital role in soil nutrient cycling [12]. Healthy soil and efficient nitrogen utilization should guarantee a high yield and efficient practices for crops. In tomato production, reduced mineral fertilizers combined with bio-organic fertilizer could improve the quality of soil and increase the quality and yield of tomato (*Solanum lycopersicum* L.) [13]. Jin et al. [14] found that reduced mineral fertilizers with biological organic fertilizers had an influence on the environment of the soil microbiota and improved the quality and output of lettuce.

Radish (*Raphanus sativus* L.), one of the main brassicaceous vegetables, plays important roles in people’s daily diet. However, excessive application of nitrogen fertilizer is also very common in the production of brassicaceous crops. The reduction of nitrogen fertilizer application is imperative. Studies found bio-organic fertilizers with 20% reduction of nitrogen significantly increased the soil organic matter content, promoted the growth and increased the economic benefit of radish [15,16]. Cai’s research suggested that nitrogen fertilizer reduced by 25% combined with bio-organic fertilizer rich in *Trichoderma* could enhance the availability of soil nutrients and increase the yield of tomato [17]. Feng’s research suggested that *Bacillus* bio-organic fertilizer significantly increased the Vc content, decreased the nitrite content, and improved the yield and quality of cauliflower [18].

Investigations have shown that the partial substitution of nitrogen fertilizer by bio-organic fertilizers increased the economic benefit of radish. However, for the diversity and continuous development of bio-organic fertilizer, there are still many areas worthy of further study. And few studies have focused on the variation of the microbial community after applying bio-organic fertilizers with reduced chemical fertilizers, especially with radish. Therefore, in this study, we used three kinds of bio-organic fertilizers with 20% reduction of chemical fertilizer, and the effects of these fertilizers on the soil characteristics and microbial community, as well as the plant yield and quality, were investigated in radish. This study will provide theoretical and practical support for the scientific application of fertilizers during the process of growing radishes.

## 2. Results

### 2.1. Soil Chemical Properties and Enzyme Activities

Nitrogen reduction by 20% with ‘No. 1’ bio-organic fertilizers increased the organic matter, pH, total nitrogen, ammonium and nitrate nitrogen of soil (Table 1). The organic matter, pH, and the whole nitrogen of N1 were significantly higher than that of T1. The ammonium nitrogen of N1 and J2 was significantly higher than in the other treatments. Soil urease and FDA enzyme activity of N1 were significantly higher than the other treatments (Figure S1). Soil sucrase enzyme activity of J2 was significantly higher than that of T1.

### 2.2. Soil Microbiomes

After quality filtering, 762,045 sequences were clustered into 2547 OTUs with the bacteria, and 1,119,924 sequences were clustered into 471 OTUs with the fungi. All the treatments with fertilizers decreased the Sobs, Shannon and ACE indices of bacteria. On the contrary, nitrogen reduction of 20% with bio-organic fertilizers increased the Shannon index of fungi compared with T1 (Table S1).

**Figure 1:**
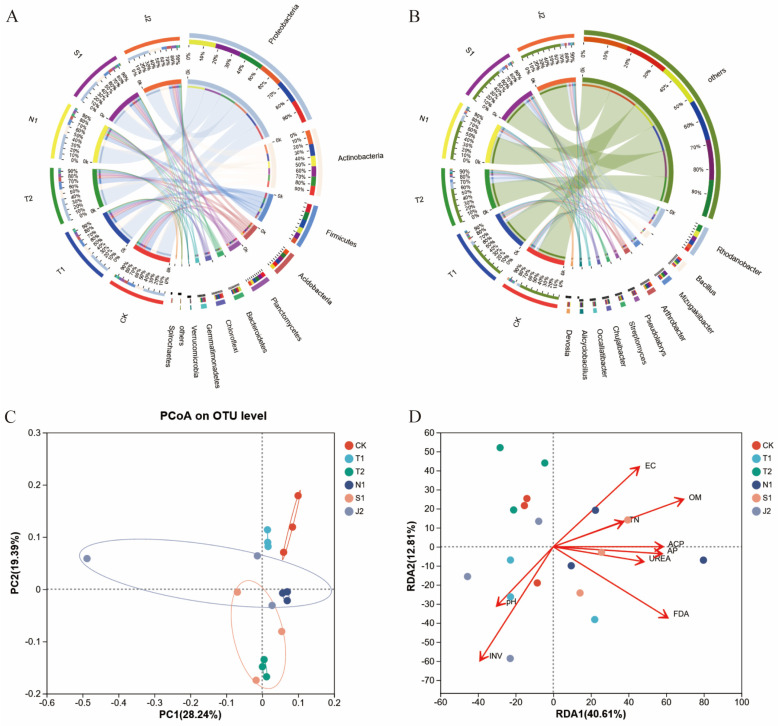
Relative abundance of bacteria (**A**) at the phylum level; relative abundance of bacteria (**B**) at the genus level; principal coordinate analysis (PCoA) plots of bacteria (**C**); redundancy analysis of dominant bacteria (**D**) associated with soil properties. Note: TN: soil total nitrogen content, OM: soil organic matter, ACP: acid phosphatase activity, AP: available phosphorus, UREA: urease activity of soil, INV: invertase activity, FDA: fluorescein diacetate activity. The same below.

**Figure 2:**
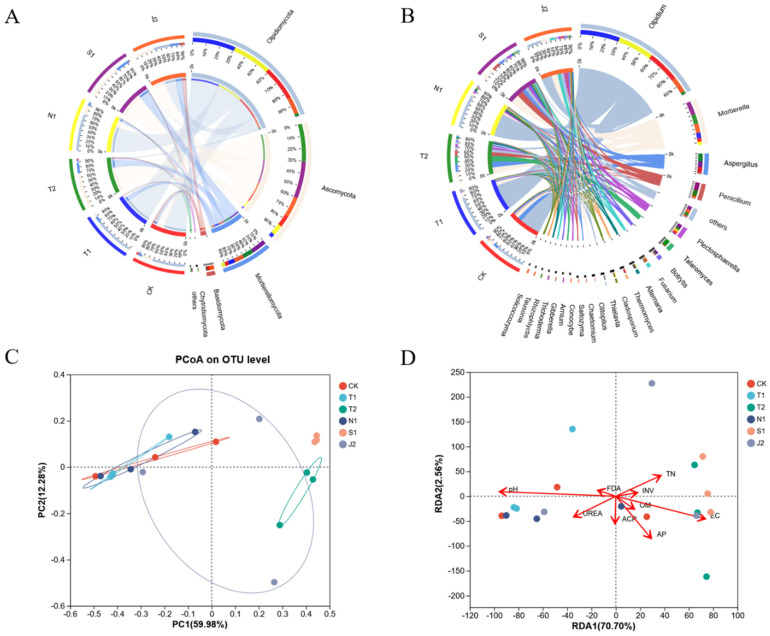
Relative abundances of fungi (**A**) at the phylum level; relative abundance of fungi (**B**) at the genus level; principal coordinate analysis (PCoA) plots of fungi (**C**); redundancy analysis of dominant fungi (**D**) associated with soil properties.

At the level of the phylum (Figure 1A and Figure 2A), the dominant bacteria in all of the soil samples were *Proteobacteria*, *Actinobacteria*, *Firmicute*, *Planctomycetes*, *Bacteroidetes*, *Chloroflex*, and *Gemmatimonadetes*. Both *Proteobacteria* and *Bacteroidetes* relative abundance in N1 were increased compared with other treatments. At the phylum level for all samples, the dominant fungi were *Olpidiomycota*, *Ascomycota*, *Basidiomycota*, *Mortierellomycota*, and *Chytridiomycota*. The *Olpidiomycota* relative abundance in N1 and T1 were both increased compared with the other treatments.

At the genus level (Figure 1B and Figure 2B), the top six dominant bacterial genera of all samples were *Rhodanobacter*, *Bacillus*, *Mizugakiibacter*, *Arthrobacter*, *Pseudolabrys*, and *Streptomyces*. Additionally, *Bacillus* and *Streptomyces* in N1 were increased compared with T1. The top six dominant fungal genera were *Olpidium*, *Mortierella*, *Aspergillus*, *Penicillium*, *Plectosphaerella*, and *Talaromyces* in all samples. *Olpidium* in N2 was increased compared with T2, S1 and J2.

The PCoA analysis showed that a reduction of mineral fertilizer combined with different bio-organic fertilizers altered the soil bacterial and fungal community composition. For the community structure of bacteria, the first and second principal coordinates explained 28.24 and 19.39% of the six treatments, respectively (Figure 1C). Moreover, CK, T2 and N1 were considerably separated from T1. For the community structure of fungi, the first and second principal coordinates explained 59.98 and 12.28% of the six treatments, respectively (Figure 2C). Moreover, T2 and S2 were considerably separated from T1.

**Figure 3:**
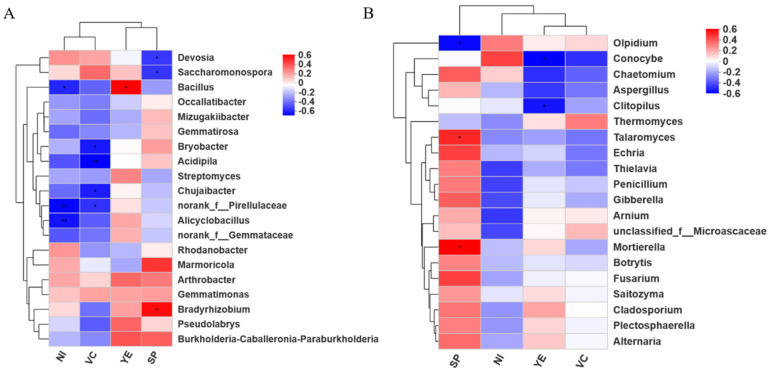
Heatmap of the correlations among the dominant (the top 20) genera associated with yield and quality. (**A**) Bacterial; (**B**) fungi. Note: * The correlation was significant at the 0.05 level (double-tailed). ** The correlation was significant at the 0.01 level (double-tailed). NI: nitrate content, VC: vitamin C content, YE: yield per plant, SP: soluble protein content.

The RDA analysis in Figure 3 shows the relationship between the microbial community composition and the soil chemical properties. The first two axes of the RDA analysis explained 40.46% and 12.81% of the total variance with the community of soil bacteria (Figure 1D). Within the factors, OM (*p* = 0.011), INV (*p* = 0.007) and FDA (*p* = 0.027) had significant influences on the structure of the bacterial community. The first two axes of the RDA analysis explained 70.70% and 2.56% of the variance in the soil fungal community. Among the factors, pH (*p* = 0.007) and EC (*p* = 0.029) had significant influences on the structure of the fungal community (Figure 2D).

Moreover, the Spearman correlation analysis results revealed the relationship with the genus level, the yield and quality of the plants (Figure 3). In the bacterial community, *Bacillus* had a significant positive correlation with yield. On the contrary, the nitrate content had a significant negative correlation with *Bacillus*. In the fungal community, the yield had a significant negative correlation with *Conocybe* and *Clitopilus*. The soluble protein content had a significant positive correlation with *Talaromyces* and *Mortierella*.

### 2.3. Photosynthetic Characteristics, Growth, Yield and Quality

**Figure 4:**
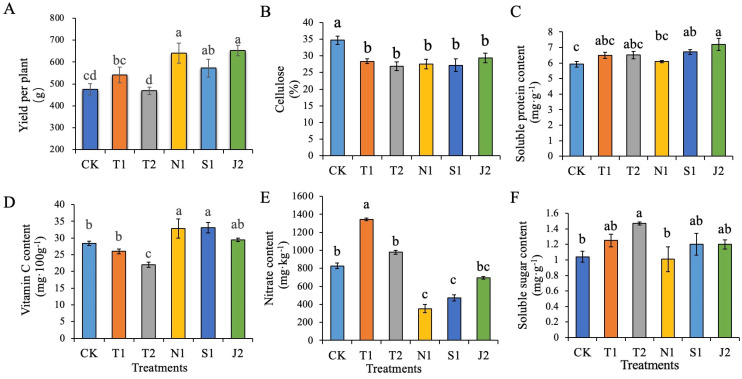
Effects of nitrogen reduced 20% combined with bio-organic fertilizer on yield and quality of radish. (**A**) Yield per plant; (**B**) cellulose; (**C**) soluble protein content; (**D**) vitamin C content; (**E**) nitrate content; (**F**) soluble sugar content. Note: Significant differences analysis was performed with Duncan’s multiple range test (*p* < 0.05) with SPSS (IBM, Chicago, IL, USA, version 22.0), column bar means standard error, different small letters represent significant differences at the 0.05 level.

In this study, we found that the chlorophyll a, chlorophyll b, and total chlorophyll content of N1 were significantly higher than that of T1 (Figure S2). The yield, plant leaf length, leaf width, crown length, plant height, and plant crown width of N1 were the highest and significantly higher than in T1 (Table S2). Except for T2 and CK, the soluble sugar contents had no significant difference among the treatments. The cellulose of CK was the highest and significantly higher than the other treatments (Figure 4). The content of soluble protein with J2 was slightly higher than CK, whereas N1, S1 and J1 had no significant differences from T1 and T2. The vitamin C contents of N1 and S1 were significantly higher than those of T1 and T2. And the contents of nitrate with N1 and S1 were significantly lower than with CK, T1 and T2.

## 3. Discussion

Soil provides the growth environment and nutrients required by plants, and fertile soil is the basis of high crop yields and good quality produce. To solve problems caused by excessive fertilization and achieve sustainable development of radish production, in this experiment, reducing the synthetic nitrogen fertilizer by 20% and supplementing with “No. 1”, “Seek” or “Jiajiapei” bio-organic fertilizers was adopted to analyze their effects on soil quality, plant yield and quality. Soil pH is the dominant factor regulating soil nutrient bioavailability and soil microbial community structure [19,20]. Organic matter alters soil microbial community activities, which are essential and can be used as an overall indicator of soil health [21]. In this experiment, supplementing synthetic nitrogen fertilizer with bio-organic fertilizer enhanced the soil chemical properties compared with the conventional fertilization (Table 1). In acidic soils, the soil pH increased from 5.87 to 6.42, 6.29 and 6.10 with bio-organic fertilizer (T2). Organic matter increased from 19.22 g·kg^−1^ to 28.09 g·kg^−1^ and 24.48 g·kg^−1^, although the effect was not obvious for “Jiajiapei”. Among them, the soil pH, organic matter and total nitrogen content of the N1 treatment (20% nitrogen reduction by full-effective “No. 1” enriched with *Bacillus* bio-organic fertilizer) were the highest and significantly higher than those under CK and T1. Silvosa et al. [15] found that nitrogen reduction of 20% with bio-organic fertilizer increased the soil organic matter content of radish. Jin et al. [14] and Qi et al. [22] also found that *Bacillus* enriched bio-organic fertilizer increased the soil pH and organic matter content for cabbage, which is similar to our results. Silvosa et al. [15] found that nitrogen reduction of 20% with bio-organic fertilizer increased the soil organic matter content for radish.

Nitrogen is an essential macronutrient for crop growth. And soil nutrient cycling is influenced by specific soil enzyme activities, which are closely related to the soil microbial community structure [23,24,25]. In this study, the results demonstrated that nitrogen reduction with *Bacillus* bio-organic fertilizer significantly improved the soil urease and FDA enzyme activity compared with conventional fertilization (T1) (Figure S1). FDA hydrolysis is widely accepted as an accurate and simple method for measuring total microbial activity in soil [26]. Yang et al. [27] found that the application of bio-organic fertilizer increased the activity of soil urease, acid phosphatase and FDA enzyme. Gou et al. [28] and Wang et al. [29] also found that the application of *Bacillus* bio-organic fertilizers increased the soil invertase and FDA enzyme activity for pepper and cotton, respectively, which were similar to our results. Moreover, their study showed that the enzyme activity was different among the three bio-organic fertilizers, which may be related to the differences in functional microbes in them [30].

Soil microbes are vital to the health of the soil, and beneficial microorganisms ultimately affect the growth and yield of plants by altering the soil microbial community structure [31,32]. Previous research showed that bio-organic fertilizer could change the soil microbial diversity [33]. The dominant bacterial and fungal phyla of soil were changed with the application of bio-organic fertilizer [34,35]. In this study, we found that the application of bio-organic fertilizer changed the soil microbial diversity, as well as the dominant bacterial and fungal phyla of the soil (Table S1, Figure 1 and Figure 2). At the phylum level (Figure 2B), the dominant bacteria were *Proteobacteria*, *Actinobacteria* and *Firmicute* in all the samples. *Proteobacteria* have the function of lignin degradation and nitrogen fixation [36]. *Bacteroidetes* have the ability to degrade macromolecular organic matter [37]. Both hold the possibility of improving soil quality. The dominant fungal phyla were *Olpidiomycota*, *Ascomycota*, and *Mortierellomycota*. *Ascomycetes* could degrade the unstable parts of organic residues [38]. And moreover, the soil microbial community composition was correlated with pH, OM, INV and FDA after bio-organic fertilizers were applied (Figure 1 and Figure 2). Fierer’s [12] research suggested that the pH of soil might be the most vital factor that has notable influences on the soil’s bacterial community structure. In our experiment, applying bio-organic fertilizers shifted the soil pH much closer to neutral and improved the soil bacterial communities.

Bio-organic fertilizer application improved soil quality, which in turn promoted the growth of radish, as well as the yield. Among all the treatments, N1 presented the highest yield, increased by12.11% compared with T1. The analysis also suggested that the yield had a significant positive correlation with *Bacillus* (Figure 4). On the contrary, the nitrate content had a significant negative correlation with yield. *Bacillus* is an important PGPR (plant growth-promoting Rhizobacteria), which can promote the absorption of nutrients and secrete plant hormones [39,40,41,42]. The activity of nitrate reductase was also increased. Maybe due to more absorption of nitrate, the nitrate content in the soil was reduced. Previous studies showed that *Bacillus* enriched bio-organic fertilizers could promote the growth of cucumber [43], pepper [44] and banana plants [45], which is similar to our results. In addition, nitrogen reduction 20% with *Bacillus* bio-organic fertilizer significantly increased the Vc content and decreased the nitrate and cellulose content of radish. These findings mean that it can improve the radish quality.

## 4. Materials and Methods

### 4.1. Materials

The seeds of radish ‘Nanlvcui’ were supplied by the National Key Laboratory of Crop Genetics and Germplasm Enhancement, College of Horticulture, Nanjing Agricultural University. The field experiments were conducted at Agricultural Expo Garden, Jurong, Jiangsu, China (32° N, 119°12′ E). The soil in the plowed layer (0–15 cm) was acid soil with a pH of 5.86; the organic matter, total nitrogen, available phosphorus and available potassium content was 28.18 g·kg^−1^, 1.54 g·kg^−1^, 212.21 mg·kg^−1^ and 178.20 mg·kg^−1^, respectively.

Bio-organic fertilizer enriched with *Bacillus* named ‘No. 1’ (*Bacillus* 2 × 10^8^·g^−1^ living bacteria count, 3-5-0.7 N-P_2_O_5_-K_2_O) was provided by Lianye Co., Ltd., Jiangyin, China. ‘Seek Bamboo Charcoal’ (biochar fertilizer, 3-5-0.7 N-P-K) was purchased from Shike Co., Ltd., Shanghai, China. Bio-organic fertilizer enriched with *Trichoderma* named ‘Jiajiapei’ (*Trichoderma* 1 × 10^9^·g^−1^ living fungi count etc., 2-2-1 N-P-K) was purchased from Delong Biotechnology Co., Ltd., Xi’an, China. Mineral fertilizers (46% urea, 12% superphosphate and 52% potassium sulfate) were obtained from Yuntianhua Co., Ltd., Kunming, China.

### 4.2. Experimental Design

Six treatments were set up in this experiment: no fertilization (CK), conventional fertilization (the average level of fertilization commonly used by farmers, T1), conventional nitrogen reduced 20% (T2), and conventional nitrogen reduced 20% with ‘No. 1’ (N1), ‘Seek’ (S1) or ‘Jiajiapei’ (J2). Each treatment was set up with three replications in a random block arrangement. The area within each plot was 6 m × 1.2 m with 150 radish plants. All of the mineral fertilizers and the Bio-organic fertilizer were applied once as a base fertilizer. The treatments are shown in Table 2. The experiment was conducted from April 2019 to June 2020, with three crop stubbles in a rotation system. Soil was collected after the third harvest in June 2020 and related indices were measured.

### 4.3. Determination Indexes and Methods

#### 4.3.1. Determination of Rhizosphere Soil Characteristics

Fresh rhizosphere soils were collected from 5 to 15 cm soil layers around the plant root for analyzing soil enzyme activity and stored at −80 °C for DNA sequencing. The pH of soil and electrical conductivity (EC) was measured by mixing soil with deionized water at 1:5 and 1:2.5 (*w*/*v*), respectively. Soil total nitrogen and organic matter content was determined by an elemental analyzer (Vario EL elemental analyzer, Hanau, Germany) [46]. The soil nitrate nitrogen content was determined by a continuous flow analyzer (BRAN + LUEBBE Auto Analyzer3, Hamburg, Germany) [47]. Available P was detected following the method of [48]. Activity of soil urease, invertase, fluorescein diacetate (FDA) and phosphatase was determined by the method described by Sun et al. [49], Taylor et al. [50] and Guan [51], respectively.

#### 4.3.2. DNA Extraction and PCR Amplification

The microbial community DNA of the rhizosphere soil was extracted from 0.5 g soil samples with an E.Z.N.A. R soil DNA Kit (Omega Bio-tek, GA, US) according to the instructions of the manufacturer. The extracted DNA was checked on a 1% agarose gel, and the DNA concentration and purity were determined by a NanoDrop 2000 UV–VIS spectrophotometer (Thermo Scientific, Wilmington, DE, USA). Soil bacterial 16S rRNA genes and fungal and protists 18S rRNA genes were studied by primer sets [52,53]. The PCR conditions are shown in Table S3 for each primer set. For each reaction, 0.4 μL of TransStart FastPfu DNA Polymerase, template DNA 10 ng, and ddH_2_O up to 20 μL were included. PCR reactions were performed in triplicate following the manufacturer’s instructions and were quantified by a Quantus™ fluorometer (Promega, Madison, WI, USA). We extracted the PCR product from the band on an agarose gel, which was purified with the AxyPrep DNA Gel Extraction Kit (Axygen Biosciences, Union City, CA, USA).

Purified amplicons were pooled in equimolar ratios and sent for paired-end sequencing on an Illumina MiSeq PE 300 × 2 sequencer (Majorbio Bio-Pharm Technology Co., Ltd., Shanghai, China). Then, we used Fastp software to analyze the raw sequences (version 0.19.6) for quality control. We dereplicated, sorted, and clustered the reads into operational taxonomic units (OTUs) at the default 97% similarity by UPARSE (version 7.0.1090), dechimerized against the UCHIME reference dataset. Taxonomic labels were assigned to the OTUs by the Silva 16S bacterial database and the UNITE fungal database (version 7.2).

#### 4.3.3. Growth, Yield and Quality

The yield and quality of radish were measured in April 2020. The plants were harvested when reaching commercial standards. The content of chlorophyll was determined by the method of ethanol (95%) extraction, colorimetric [54]. Nitrate content was analyzed by salicylic acid, colorimetric, which was determined at the 410 nm wavelength by using an atomic absorption spectrophotometer (SP-3800 Shanghai China) [55]. The Vc (L-ascorbic acid) content was measured with the anthrone colorimetry method, which was determined at the 525 nm wavelength; the soluble protein content was detected by the Coomassie Brilliant Blue G-250 colorimetric method at the 595 nm wavelength; and total soluble sugar content was measured by an o-phenanthroline colorimetric method at the 625 nm wavelength [56].

### 4.4. Statistical Analysis

Spearman’s rank correlation analysis and redundancy analysis (RDA) were used for analyzing the relationship between soil environmental factors and microbial communities. The effects of different treatments on the radish yield, quality and soil chemical properties were evaluated by one-way analysis of variance (ANOVA). Significant differences analysis was performed with Duncan’s multiple range test (*p* < 0.05) with SPSS (IBM, Chicago, IL, USA, version 22.0). The bar charts were drawn by using office software 2016. The characteristics of alpha-diversity in each soil sample were calculated by QIIME 2. Venn diagrams were obtained with the tool of Venny.

## 5. Conclusions

Nitrogen reduction 20% with *Bacillus* bio-organic fertilizer is an effective way to avoid the overuse of mineral fertilizers. It could regulate soil pH and increase the organic matter content, soil sucrase and FDA enzyme activity. RDA analysis showed that the soil AP, OM, INV and FDA were significantly correlated with the structure of the microbial community. With a good soil, the yield and vitamin C in radish increased, whereas the nitrate and cellulose content of radish decreased. The yield had a significant positive correlation with *Bacillus*. On the contrary, the nitrate content had a significant negative correlation with *Bacillus*.

## Figures and Tables

**Table 1:** Effects of nitrogen reduction combined with bio-organic fertilizer on soil chemical properties of radish.

Treatments	pH	EC ms·m^−1^	Organic Matter g·kg^−1^	Available Phosphorus g·kg^−1^	Total Nitrogen g·kg^−1^	Ammonium Nitrogen g·kg^−1^	Nitrate Nitrogen g·kg^−1^
CK	6.10 ± 0.02 c	116.07 ± 18.08 c	18.50 ± 0.71 b	16.78 ± 2.6 ab	1.84 ± 0.30 c	1.00 ± 0.47 c	20.30 ± 1.76 ab
TI	5.87 ± 0.02 d	165.07 ± 27.95 bc	19.22 ± 1.78 b	19.1 ± 1.24 ab	2.26 ± 0.49 b	0.39 ± 0.11 d	18.30 ± 1.31 b
T2	6.05 ± 0.02 c	238.00 ± 11.14 b	23.25 ± 0.21 ab	20.22 ± 3.4 ab	2.35 ± 0.24 b	1.83 ± 1.27 b	23.70 ± 2.11 a
N1	6.42 ± 0.03 a	225.33 ± 5.55 b	28.09 ± 0.66 a	22.64 ± 0.74 a	2.65 ± 0.28 a	2.51 ± 0.80 a	23.50 ± 0.8 a
S1	6.29 ± 0.05 b	310.00 ± 6.08 a	24.48 ± 3.78 ab	25.69 ± 0.93 a	2.35 ± 0.17 b	0.94 ± 1.27 c	18.65 ± 1.48 b
J2	6.10 ± 0.02 c	105.53 ± 9.90 c	17.97 ± 0.85 b	13.56 ± 1.15 b	2.41 ± 1.06 b	2.9 ± 1.02 a	20.36 ± 0.25 ab

Note: CK, no fertilization; T1, 100% fertilization; T2, 20% nitrogen reduction; N1, 20%nitrogen reduction + 1500 kg·ha^−1^ ‘No. 1’; S1, 20% nitrogen reduction + 1500 kg·ha^−1^ ‘Seek’; J2, 20% nitrogen reduction + 1080 kg·ha^−1^ ‘Jiajiapei’. The values in the table are the average of three repetitions. In each column, different small letters means significant difference at 0.05 level by Duncan’s test; the same below.

**Table 2:** Different fertilization treatments.

Treatments	Mineral Fertilizer			Bio-Organic Fertilizer		
	N kg·ha^−1^	P_2_O_5_ kg·ha^−1^	K_2_O kg·ha^−1^	NO. 1 kg·ha^−1^	Seek kg·ha^−1^	Jiajiapei kg·ha^−1^
No fertilizer (CK)	—	—	—	—	—	—
Conventional fertilizer (T1)	168	150	195	—	—	—
80% N (T2)	134.4	150	195	—	—	—
80% N + 20% NO. 1 (N1)	89.4	75	184.5	1500	—	—
80% N + 20% Seek (S2)	89.4	75	184.5	—	1500	—
80% N + 20% Jiajiapei (J2)	120.8	128.4	184.2	—	—	1080

## Data Availability

Data is contained within the article and Supplementary Materials.

## Supplementary Materials

The following supporting information can be downloaded at: https://www.mdpi.com/article/10.3390/plants14091389/s1, Figure S1: Soil-enzyme activities in the soil of radish under different fertilization treatments; Figure S2: Effects of nitrogen reduced 20% combined with bio-organic fertilizer on leaf photosynthetic pigment content of radish; Table S1: Effect of different fertilizer treatments on bacterial and fungi alpha diversity indexes of soil; Table S2: Effects of bio-organic fertilizer with nitrogen reduction on growth of radish; Table S3: Primers used in this study.
