# Supplementary material for: Bacillus Bio-Organic Fertilizer Altered Soil Microorganisms and Improved Yield and Quality of Radish (Raphanus sativus L.)
Source: Plants (Basel). 2025 May 5;14(9):1389. doi: 10.3390/plants14091389 (PMC12073825; doi:10.3390/plants14091389)
Supplement: Supplementary file 1 [file plants-14-01389-s001.zip › plants-3594870-supplementary.pdf]

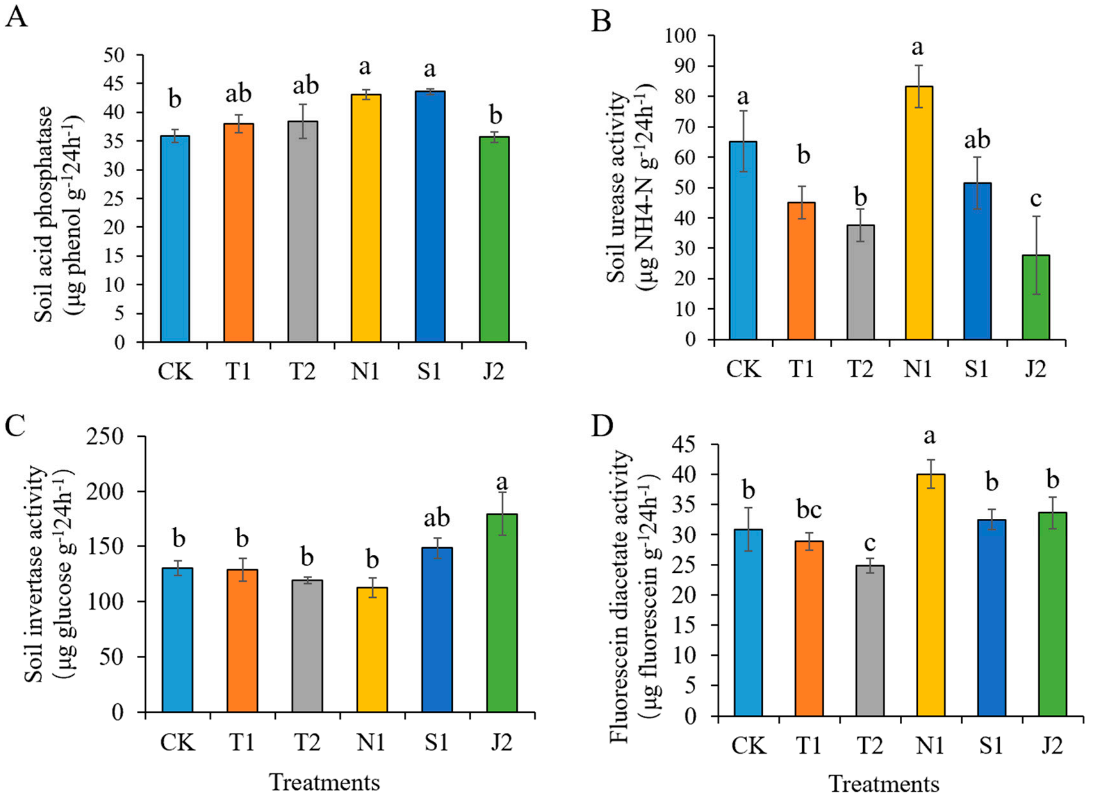

Figure S1 Soil-enzyme activities in the soil of radish under different fertilization treatments
A: Soil acid phosphatase activity; **B:** Urease activity; **C:** Invertase activity; **D:** Fluorescein diacetate activity

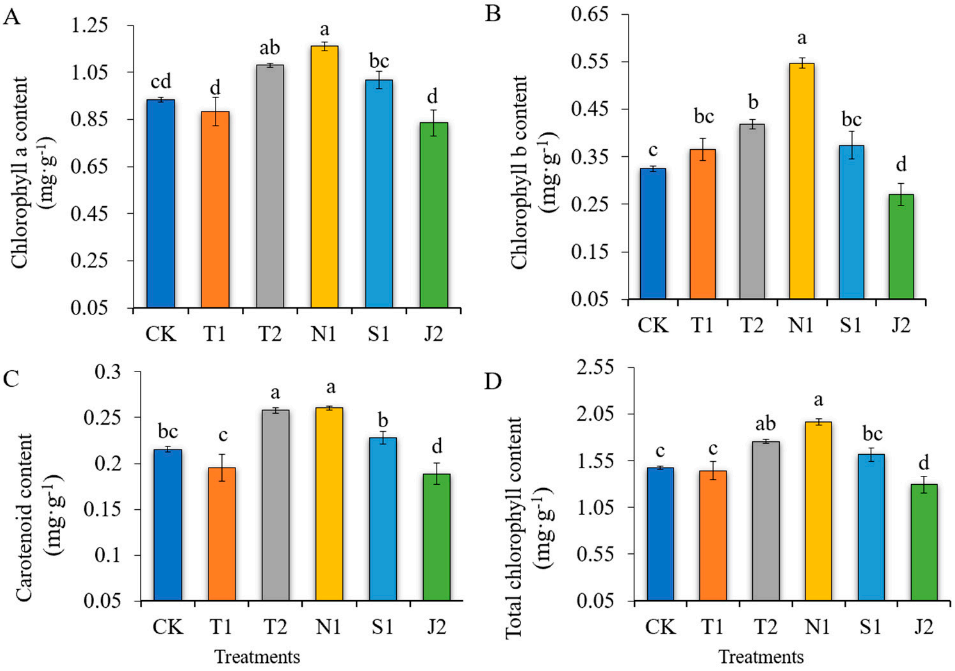

Figure S2 Effects of nitrogen reduced 20% combined with bio-organic fertilizer on leaf photosynthetic pigment content of radish; **A:** Chlorophyll a content; **B:** Chlorophyll b content; **C:** Carotenoid content**; D: T**otal Chlorophyll content

Table S1. Effect of different fertilizer treatments on bacterial and fungi alpha diversity indexes of soil

	Treatment	Coverage	Sobs	Shannon	Simpson	ACE
Bacterial	CK	0.98	1840.33±46.25a	6.11±6.11a	0.006±0.006c	2440.14±2440a
	T1	0.98	1725.00±9.00ab	6.01±6.01a	0.006±0.006bc	2287.49±2287.49ab
	T2	0.98	1394.67±13.69d	5.65±5.65c	0.010±0.010a	1891.39±1891.39c
	N1	0.98	1640.33±62.39bc	5.93±5.93ab	0.008±0.008abc	2194.78± 2194.78abc
	S1	0.98	1489.33±47.25cd	5.77±5.77bc	0.009±0.009ab	2064.46±2064.46ab
	J2	0.98	1432.67±111.68d	5.69±5.69bc	0.009±0.009ab	1936.96±1936.96ab
Fungi	CK	0.99	202.33±55.18b	1.46±0.67b	0.525±0.23ab	278.96±50.63b
	T1	0.99	185.00±20.66b	0.98±0.35b	0.670± 0.14ab	249.25±24.71b
	T2	0.99	301.33±20.33a	3.28±0.30a	0.094±0.04b	368.29±31.51a
	N1	0.99	171.00±16.04b	1.24±0.44b	0.575±0.18a	242.07±20.29b
	S1	0.99	310.00±6.93a	3.29±0.09a	0.080±0.01b	367.67±2.63a
	J2	0.99	257.00±15.39ab	2.30±0.40ab	0.302±0.11ab	310.77±2.99ab

Table S2. Effects of bio-organic fertilizer with nitrogen reduction on growth of radish

Treatments	Plant height (cm)	Leaf length (cm)	Leaf width (cm)	Crown length (cm)	Crown width (cm)
CK	52.67±1.41c	49.33±1.13d	17.67±0.47d	57.33±1.60b	51.00±1.07c
T1	55.89±0.72bc	53.33±0.29bc	19.33±0.37bc	63.56±0.75a	56.89±1.07ab
T2	55.78±1.36bc	52.78±1.12c	18.67±0.28cd	63.78±1.20a	54.22±1.37bc
N1	59.78±0.76 a	57.22±0.76a	21.22±0.55a	67.11±1.17a	60.00±1.80a
S1	57.67±1.24ab	56.11±0.93ab	20.56±0.71ab	66.44±0.73a	58.78±1.91a
J2	56.11±1.06b	54.56±1.07abc	20.22±0.43ab	65.78±1.22a	57.44±1.69ab

Table S3. Primers used in this study

	Primers	Primer sequence (5′- 3′)	Target gene	Target subfragment	Subfragment length (bp)	PCR reaction condition
Bacteria	FW-F515	GTGCCAGC(A/C)GCCGCGGTAA	16S	V4	291	an initial denaturation at 95 °C for 3 minutes, followed by 27 cycles of 30 s at 95 °C, annealing for 30 s at 55 °C and elongation for 45 s at 72 °C, the last step being extension at 72 °C for 10 minutes.
	REV-R806	GGACTAC(G/A/C)(G/C)GGGTATCTAAT				
Fungi	FW-F817	TTAGCATGGAATAAT(A/G)(A/G)AATAGGA	18S	V5-V7	379	an initial denaturation at 95 °C for 3 minutes, followed by 35 cycles of 30 s at 95 °C, annealing for 30 s at 55 °C and elongation for 45 s at 72 °C, the last step being extension at 72 °C for 10 minutes
	REV-R1196	TCTGGACCTGGTGAGTTTCC				
